# Comparison of the efficacy of antidiabetic agents in type 2 diabetes with MASLD: a network meta-analysis

**DOI:** 10.3389/fendo.2025.1659740

**Published:** 2026-01-07

**Authors:** Xin Shao, Jun You, Hui Zhang, XianHong Jiang

**Affiliations:** 1 Department of Pharmacy, The People’s Hospital of Leshan, Leshan, China; 2 Department of Gastrointestinal Surgery, The People’s Hospital of Leshan, Leshan, China

**Keywords:** antidiabetic agents, liver, MASLD, network meta-analysis, type 2 diabetes mellitus

## Abstract

**Objective:**

To evaluate and compare the efficacy of different antidiabetic agents in patients with metabolic dysfunction-associated steatotic liver disease (MASLD) and type 2 diabetes mellitus (T2DM).

**Methods:**

We systematically searched PubMed, Embase, the Cochrane Library, and Web of Science for relevant randomized controlled trials up to April 20, 2025. Primary outcomes were alanine aminotransferase (ALT), aspartate aminotransferase (AST), and triglycerides. Secondary outcomes included HDL, LDL, fasting plasma glucose (FPG), body mass index (BMI), and glycated hemoglobin (HbA1c). All statistical analyses were performed using R version 4.5.0.

**Results:**

A total of 21 randomized controlled trials were included, covering 15 antidiabetic agents and involving 1,717 participants. Ertugliflozin was the most effective in reducing ALT and AST levels, followed by pioglitazone and metformin for ALT, and pioglitazone and ipragliflozin for AST. Ertugliflozin also showed favorable effects on triglycerides, BMI, HDL, LDL, and liver stiffness. However, most comparisons did not reach statistical significance.

**Conclusion:**

Ertugliflozin appears to be among the most effective treatment options for improving liver function and metabolic parameters in patients with MASLD and T2DM. However, these findings should be interpreted with caution, and further high-quality studies are needed to confirm these results. Ertugliflozin may be the most effective option for improving liver function and metabolic parameters in patients with MASLD and T2DM. Further studies are needed to confirm these findings.

## Introduction

Metabolic dysfunction-associated steatotic liver disease (MASLD), which officially replaced the term non-alcoholic fatty liver disease (NAFLD) in June 2023, is also referred to as Metabolic-associated fatty liver disease (MAFLD) (1). It is a liver disease closely associated with metabolic dysfunction, characterized by the accumulation of fat in the liver along with abnormal liver function. The incidence of MASLD is increasing due to factors such as metabolic syndrome, insulin resistance, and obesity. The global prevalence is estimated to be around 30% (2). Particularly in patients with type 2 diabetes (T2DM), the comorbidity rate of MASLD is high. Studies indicate that approximately 70% of diabetic patients have MASLD (3, 4).

Despite the complex etiology of MASLD, pharmacological options specifically targeting the disease have long been limited, and management has traditionally relied primarily on lifestyle interventions, including dietary modification and increased physical activity (5, 6). Recently, however, resmetirom and semaglutide have been approved for use in patients with MASLD and F2–F3 fibrosis, representing an important advancement in therapeutic development. The pivotal ESSENCE phase 3 trial demonstrated that semaglutide significantly improved liver histology and biochemical markers in patients with noncirrhotic MASLD, marking a milestone in targeted therapy for this condition (7). Similarly, tirzepatide, a dual GLP-1/GIP receptor agonist, has shown promising effects on hepatic fat reduction, glycemic control, and weight loss in recent clinical studies (8). For patients with MASLD coexisting with T2DM, antidiabetic medications remain the cornerstone of treatment, as glucose-lowering agents can exert additional metabolic and hepatic benefits.

The antidiabetic medications commonly used in clinical practice include thiazolidinediones (TZDs), sodium-glucose co-transporter 2 inhibitors (SGLT-2 inhibitors), dipeptidyl peptidase-4 inhibitors (DPP-4 inhibitors), glucagon-like peptide-1 receptor agonists (GLP-1RAs), GLP-1 receptor/glucagon receptor (GLP-1R/GCGR) dual agonists, GLP-1 receptor/gastric inhibitory polypeptide receptor/glucagon receptor (GLP-1R/GIPR/GCGR) triple agonists, sulfonylureas, metformin, and insulin. These antidiabetic agents regulate blood glucose through various mechanisms, such as improving insulin sensitivity, promoting insulin secretion, and delaying gastric emptying, while also reducing the severity of fatty liver (9, 10).

However, the metabolic and hepatic effects of these agents differ substantially in patients with MASLD. Thiazolidinediones (e.g., pioglitazone) have been shown to be most effective in reducing the nonalcoholic fatty liver disease activity score (11). In contrast, SGLT2 inhibitors and GLP-1 receptor agonists not only lower HbA1c levels but also promote weight loss, making them particularly advantageous for MASLD management (12, 13). DPP-4 inhibitors and metformin, on the other hand, exert moderate effects on liver enzymes and glycemic control, with limited influence on liver histology (14). Despite these distinctions, few studies have systematically compared the relative efficacy of different antidiabetic agents in patients with both T2DM and MASLD. Most existing research focuses on pairwise comparisons between specific drugs, and comprehensive evidence remains lacking. Therefore, this study aims to conduct a network meta-analysis to evaluate and compare the efficacy of multiple antidiabetic agents in patients with type 2 diabetes mellitus (T2DM) and metabolic dysfunction–associated steatotic liver disease (MASLD), providing a more robust evidence base for clinical decision-making.

## Methods

This systematic review and meta-analysis was conducted in strict accordance with the Preferred Reporting Items for Systematic Reviews and Meta-Analyses for Network Meta-Analysis (PRISMA-NMA) guidelines. This network meta-analysis has been registered in PROSPERO (CRD420251037400). A completed PRISMA-NMA checklist is available in the **Supplementary Material**.

### Search strategy

We conducted a literature search across multiple databases, including PubMed, Embase, the Cochrane Library, and Web of Science, targeting randomized controlled trials (RCTs) published before April 10, 2025. The search strategy utilized key terms such as “MASLD,” “steatotic liver disease,” “MASH,” “Type 2 Diabetes Mellitus,” and specific hypoglycemic agents like “Dapagliflozin” and “Glimepiride.” Details of the full search methodology are provided in **Supplementary File Supplementary Table S1**. To enhance the comprehensiveness of our search, we also reviewed the reference lists of all included articles.

### Eligibility criteria

Criteria for inclusion (1): The study was a randomized controlled trial (RCT) (2); The participants were adult patients (aged >18 years) diagnosed with type 2 diabetes mellitus (T2DM) and metabolic associated fatty liver disease (MASLD), based on liver biopsy histology, imaging techniques, or blood biomarkers/scores (3); The intervention included thiazolidinediones (TZDs), sodium-glucose co-transporter 2 inhibitors (SGLT-2 inhibitors), dipeptidyl peptidase-4 inhibitors (DPP-4 inhibitors), glucagon-like peptide-1 receptor agonists (GLP-1RAs), GLP-1 receptor/glucagon receptor (GLP-1R/GCGR) dual agonists, GLP-1 receptor/gastric inhibitory polypeptide receptor/glucagon receptor (GLP-1R/GIPR/GCGR) triple agonists, sulfonylureas, metformin, insulin, diet therapy, or lifestyle modifications (4).; The primary outcomes included alanine aminotransferase (ALT), aspartate aminotransferase (AST), and related liver function biomarkers.

### Study selection

The literature search was independently conducted by two researchers (Shao X and Jiang XY). After duplicates were removed, studies irrelevant to the topic were excluded based on title and abstract screening. The full texts of the remaining studies were then retrieved and carefully evaluated for inclusion eligibility. In cases of disagreement during the selection process, a third researcher was consulted to reach a final consensus.

### Data extraction and outcome measures

A pre-designed table was used to collect relevant data, including the first author’s name, country, year of publication, sample size, interventions, and reported endpoints of interest. The primary outcomes were aspartate aminotransferase (AST) and alanine aminotransferase (ALT), while the secondary outcomes included liver stiffness measurement (LSM) in kPa, triglycerides, body mass index (BMI), high-density lipoprotein (HDL), low-density lipoprotein (LDL), fasting plasma glucose (FPG), and hemoglobin A1c (HbA1c). All of these steps were independently performed by two individuals (Shao X and Jiang XY), with any discrepancies resolved through discussion.

### Risk of bias assessment

Two reviewers (Shao X and Jiang XY) independently assessed the risk of bias in each included randomized controlled trial using the Cochrane Collaboration’s Risk of Bias 2 (RoB 2) tool for randomized trials. The tool evaluates five domains: randomization process (D1), deviations from intended interventions (D2), missing outcome data (D3), measurement of the outcome (D4), and selection of the reported result (D5). Each domain was judged as “low risk,” “some concerns,” or “high risk,” and the overall risk of bias was summarized accordingly. The assessments were performed using the official Excel-based RoB 2 template. Discrepancies were resolved through discussion or arbitration by a third author.

### Statistical analysis

A Bayesian network meta-analysis was performed using Markov chain Monte Carlo (MCMC) simulation. Four Markov chains were run with 50,000 iterations after a burn-in of 10,000 iterations, and convergence was assessed using trace plots and the Gelman–Rubin diagnostic (R-hat < 1.05 indicating satisfactory convergence). A consistency model was used as the primary analysis, and network consistency was evaluated using the node-splitting approach, which compares direct and indirect evidence for each comparison. Between-study heterogeneity was estimated using the τ² statistic, and overall heterogeneity was examined using the I² statistic for each outcome. The transitivity assumption was assessed by examining the similarity of clinical and methodological characteristics across the included trials, including baseline disease severity, age, BMI, and diabetes duration. Vague non-informative priors were used for treatment effects and heterogeneity parameters to minimize prior influence. All analyses were performed using R 4.5.0 with the gemtc and rjags packages.

## Results

### Literature search

**Figure 1:**
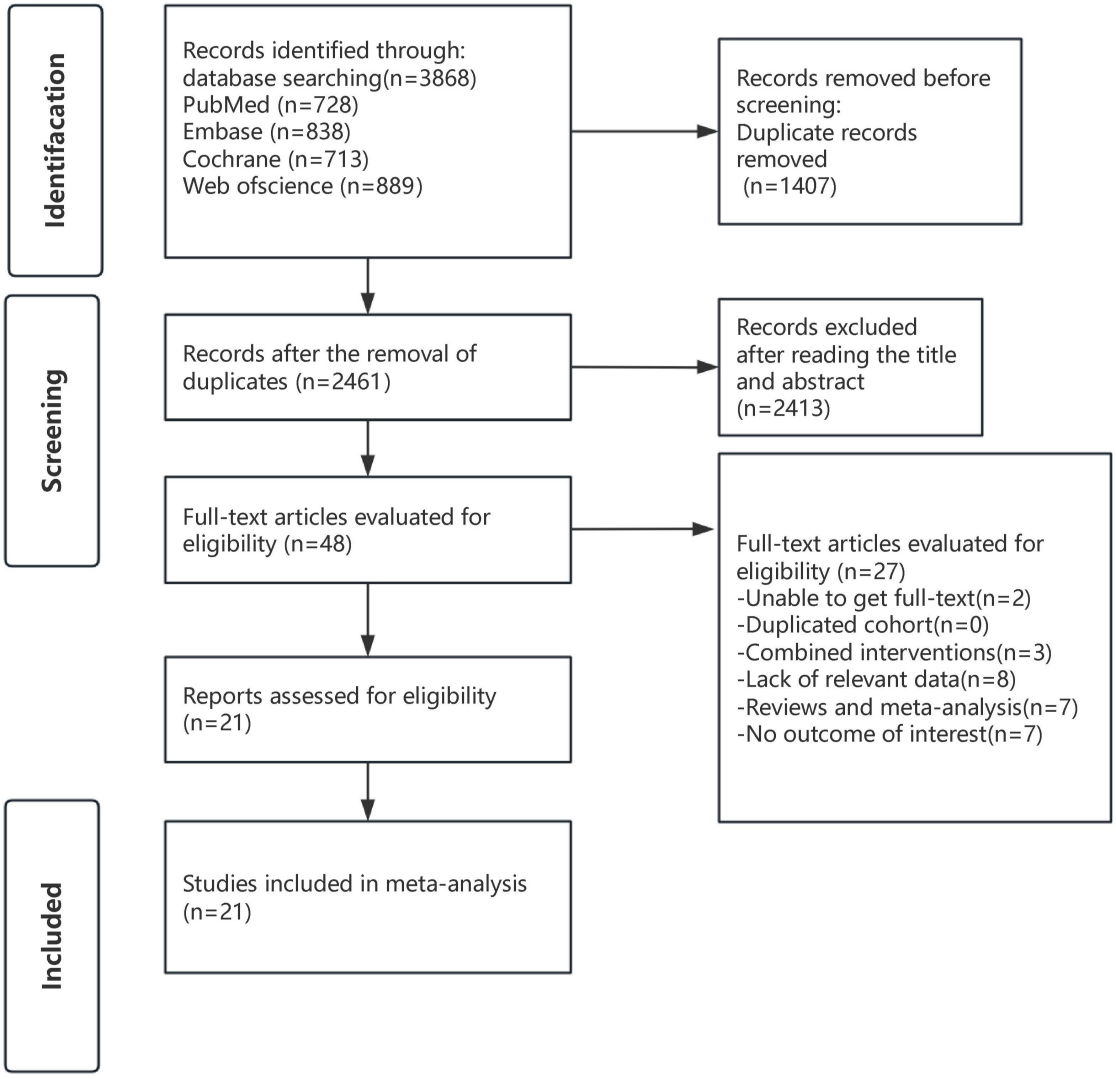
Search strategy diagram.

A comprehensive search was conducted for studies published before April 10, 2025, resulting in 3,868 records. After removing duplicates, 2,461 records were left. Initial screening based on titles and abstracts led to the exclusion of 2,413 records. The full texts of the remaining 48 articles were reviewed in depth, with 27 studies being excluded for reasons outlined in **Supplementary File Table S2**. In the end, 21 studies were included in this meta-analysis. The screening process is shown in **Figure 1**.

### Study characteristics

A total of 21 RCTs (15–35) were included in this study, originating from seven countries: Japan (n=6), China (n=5), Iran (n=3), Pakistan (n=1), India (n=1), Egypt (n=1), and the Netherlands (n=1). These studies encompassed a total of 1,717 participants and involved 15 different antidiabetic agents, including Pioglitazone (8 studies), Empagliflozin (4 studies), Liraglutide (4 studies), Dapagliflozin (4 studies), Metformin (3 studies), Sitagliptin (2 studies), Tofogliflozin (2 studies), Glimepiride (2 studies), Gliclazide (2 studies), Exenatide (2 studies), Ertugliflozin (1 study), Rybelsus (1 study), Ozempic (1 study), Ipragliflozin (1 study), and Insulin (1 study). All included studies utilized alanine aminotransferase (ALT) and aspartate aminotransferase (AST) levels as outcome measures. The characteristics of the included studies are summarized in **Table 1**.

**Table 1:** Baseline characteristics of the randomized controlled trials included in the network meta-analysis.

Study	Country	Sample	Follow-up time	Experimental group	Control group
Shojaei et al.(2024)[30]	Iran	119	24 weeks	Empagliflozin	Placebo
Khaliq et al.(2024)[26]	Pakistan	180	24 weeks	Ertugliflozin/Pioglitazone	Placebo
Gad et al.(2024)[23]	Egypt	180	24 weeks	Rybelsus/Ozempic	Placebo
Hiruma et al.(2023)[24]	Japan	42	12 weeks	Empagliflozin	Sitagliptin
Takeshita et al.(2022)[32]	Japan	40	48 weeks	Tofogliflozin	Glimepiride
Yoneda et al.(2021)[34]	Japan	38	24 weeks	Pioglitazone	Tofogliflozin
Chehrehgosha et al.(2021)[18]	Iran	106	24 weeks	Empagliflozin/Pioglitazone	Placebo
Ito et al.(2017)[25]	Japan	66	24 weeks	Pioglitazone	Ipragliflozin
Fan et al.(2013)[20]	China	117	12 weeks	Exenatide	Metformin
Feng et al.(2019)[21]	China	85	24 months	Liraglutide/Gliclazide	Metformin
Shimizu et al.(2019)[29]	Japan	63	24 weeks	Dapagliflozin	Placebo
Smits et al.(2016)[31]	the Netherlands	51	12 weeks	Liraglutide/Sitagliptin	Placebo
Tian et al.(2018)[33]	China	127	12 weeks	Liraglutide	Metformin
Aso et al.(2019)[15]	Japan	57	24 weeks	Dapagliflozin	Placebo
Attaran et al.(2023)[16]	Iran	73	24 weeks	Pioglitazone	Empagliflozin
Kinoshita et al.(2020)[20]	Japan	98	28 weeks	Pioglitazone/Dapagliflozin	Glimepiride
Zhang et al.(2020)[35]	China	60	24 weeks	Liraglutide	Pioglitazone
Feng et al.(2017)[22]	China	87	24 weeks	Liraglutide/Gliclazide	Metformin
Cho et al.(2021)[19]	Japan	53	24 weeks	Dapagliflozin	Pioglitazone
Kuchay et al.(2018)[28]	India	42	20 weeks	Empagliflozin	Placebo
Bi et al.(2014)[17]	China	33	24 weeks	Exenatide/Insulin	Pioglitazone

### Assessment of quality of included studies

**Figure 2:**
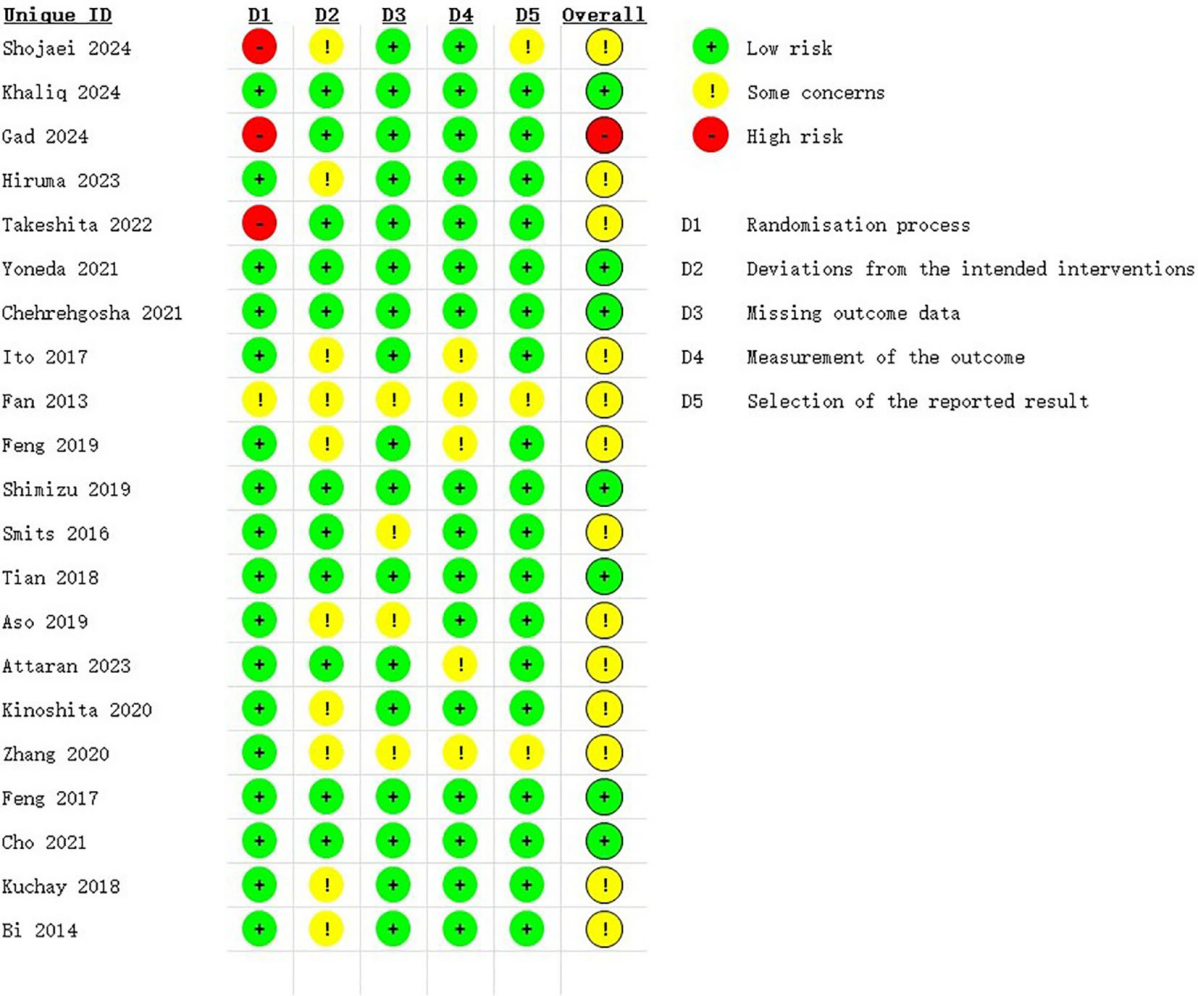
Risk of bias summary assessed with RoB2: review authors’ judgements about each risk of bias item for each included study in this trial.

**Figure 3:**
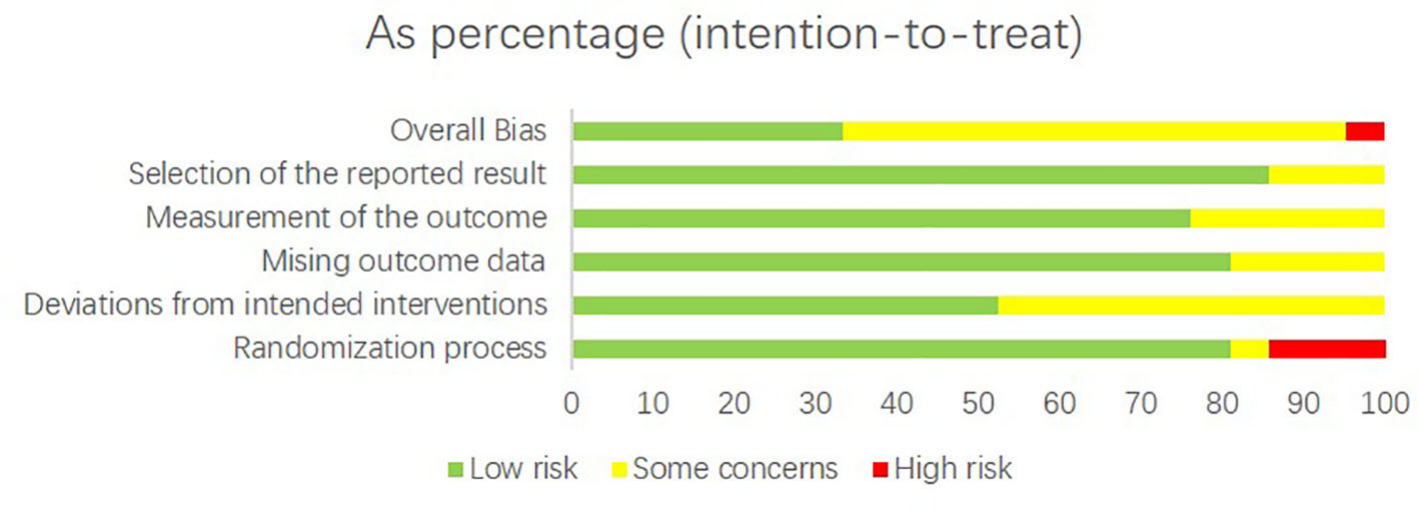
Risk of bias graph assessed with RoB2: review authors’ judgement about each risk of bias item presented as percentages across all included studies in this trial.

To assess the quality of the included studies, the Cochrane Risk of Bias 2 (RoB2) tool was applied. The results showed that 7 RCTs were rated as having a low risk of bias, 13 RCTs were judged to have some concerns, and only 1 RCT was assessed as having a high risk of bias. Detailed results are presented in **Figures 2**, **3**.

### Network meta-analysis

**Figure 4:**
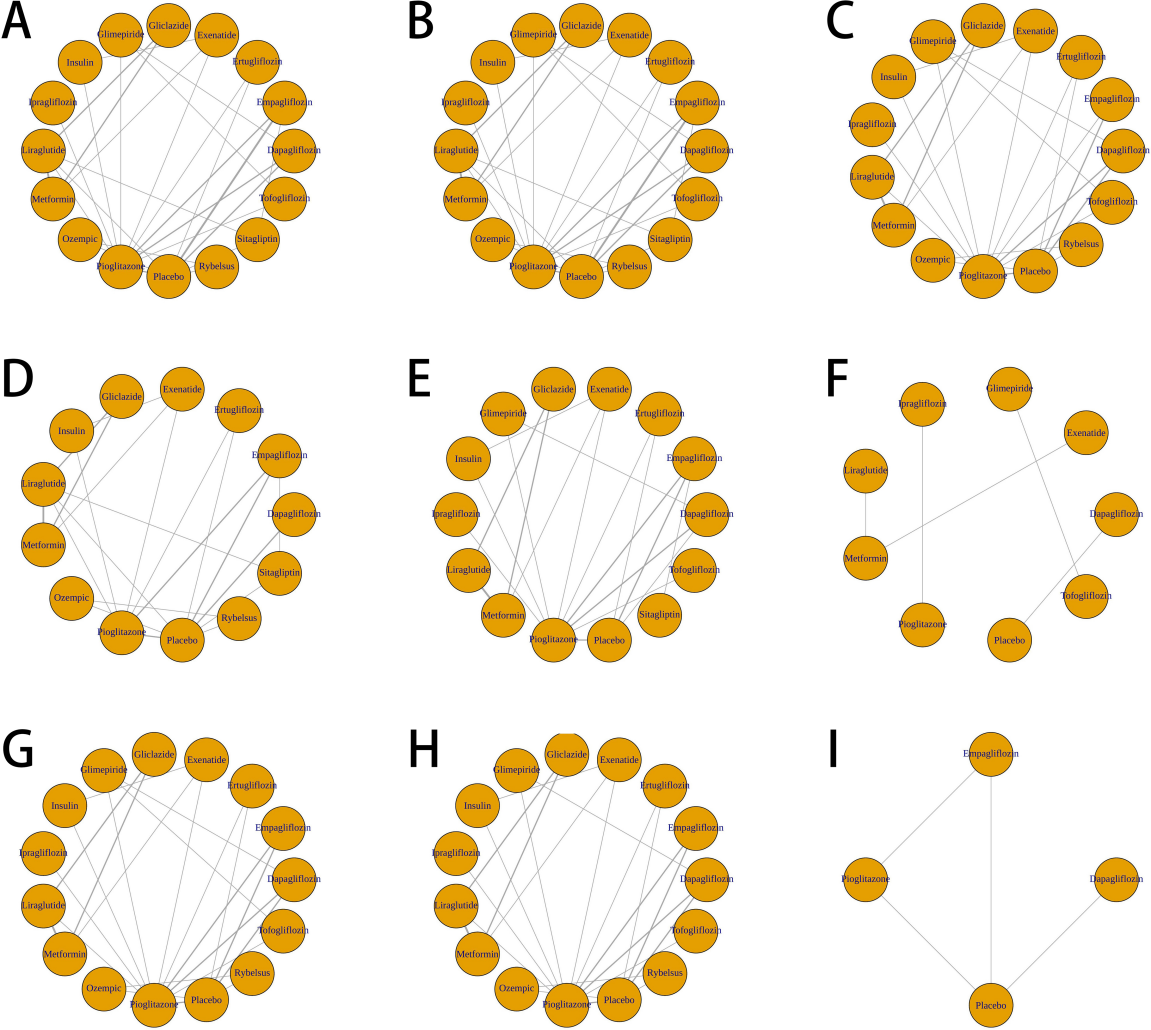
Network meta-analysis diagrams of various antidiabetic agents evaluated for multiple outcomes in patients with type 2 diabetes and MASLD. **(A)** Network plot of ALT outcome. **(B)** Network plot of AST outcome. **(C)** Network plot of triglyceride outcome. **(D)** Network plot of BMI outcome. **(E)** Network plot of FPG outcome. **(F)** Network plot of HbA1c outcome. **(G)** Network plot of HDL outcome. **(H)** Network plot of LDL outcome. **(I)** Network plot of LSM outcome.

All the included trials were incorporated into the network analysis, and the network meta-analysis diagram (**Figure 4**) illustrates the comparative effects of various antidiabetic agents on both primary and secondary outcomes in patients with type 2 diabetes and MASLD. For the primary outcomes (ALT and AST), each antidiabetic agent had available data from relevant studies. However, for some secondary outcomes such as FPG and liver stiffness measurement (LSM), network estimates could not be generated due to the limited number of eligible trials reporting these outcomes.

#### ALT

**Figure 5:**
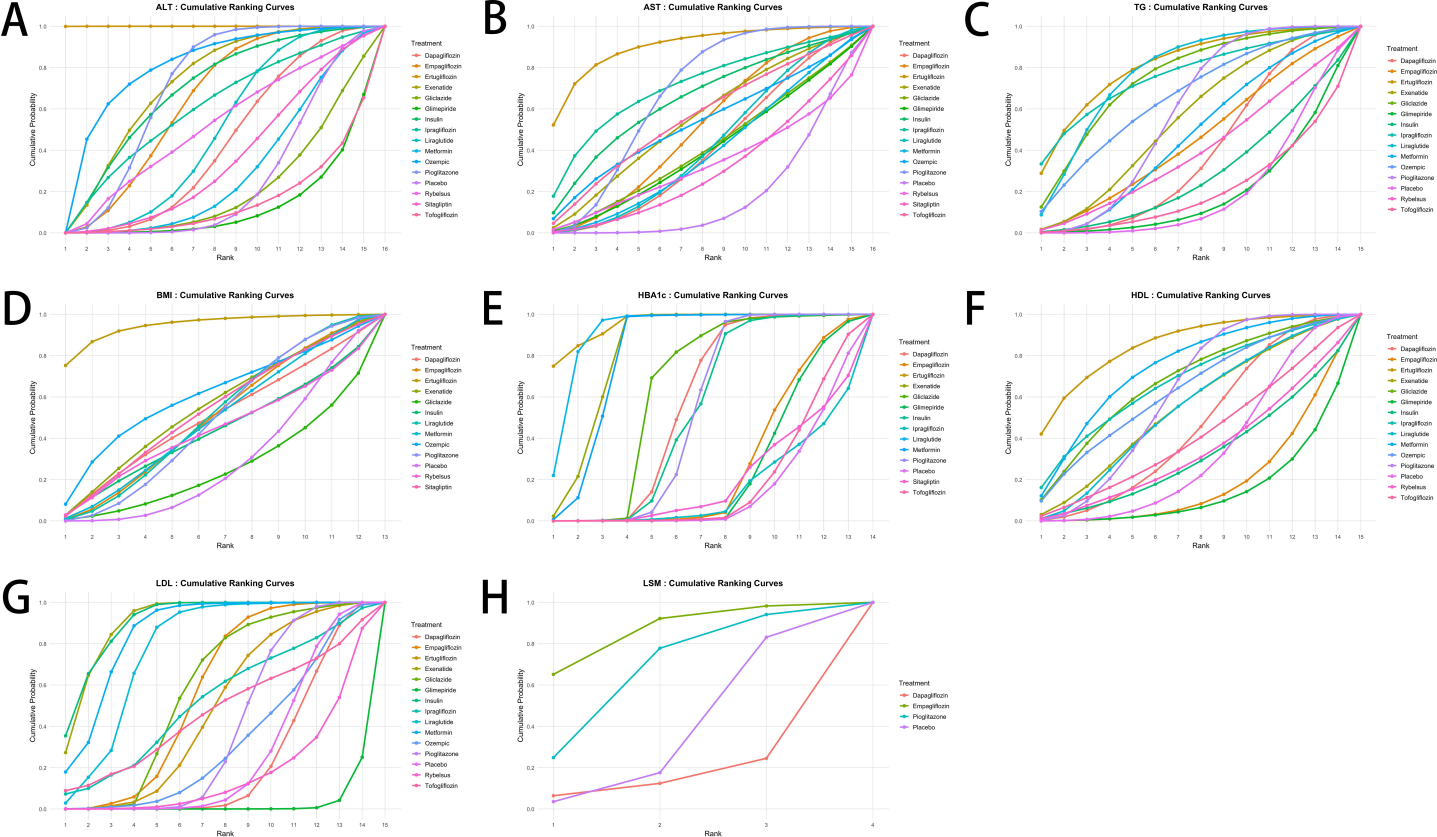
Surface under the cumulative ranking curve (SUCRA) plots for the ranking probabilities of antidiabetic agents across outcomes. **(A)** ALT; **(B)** AST; **(C)** Triglycerides; **(D)** BMI; **(E)** HbA1c; **(F)** HDL; **(G)** LDL; **(H)** LSM. Higher SUCRA values indicate a greater probability of being ranked as the most effective treatment among the compared interventions.

**Figure 6:**
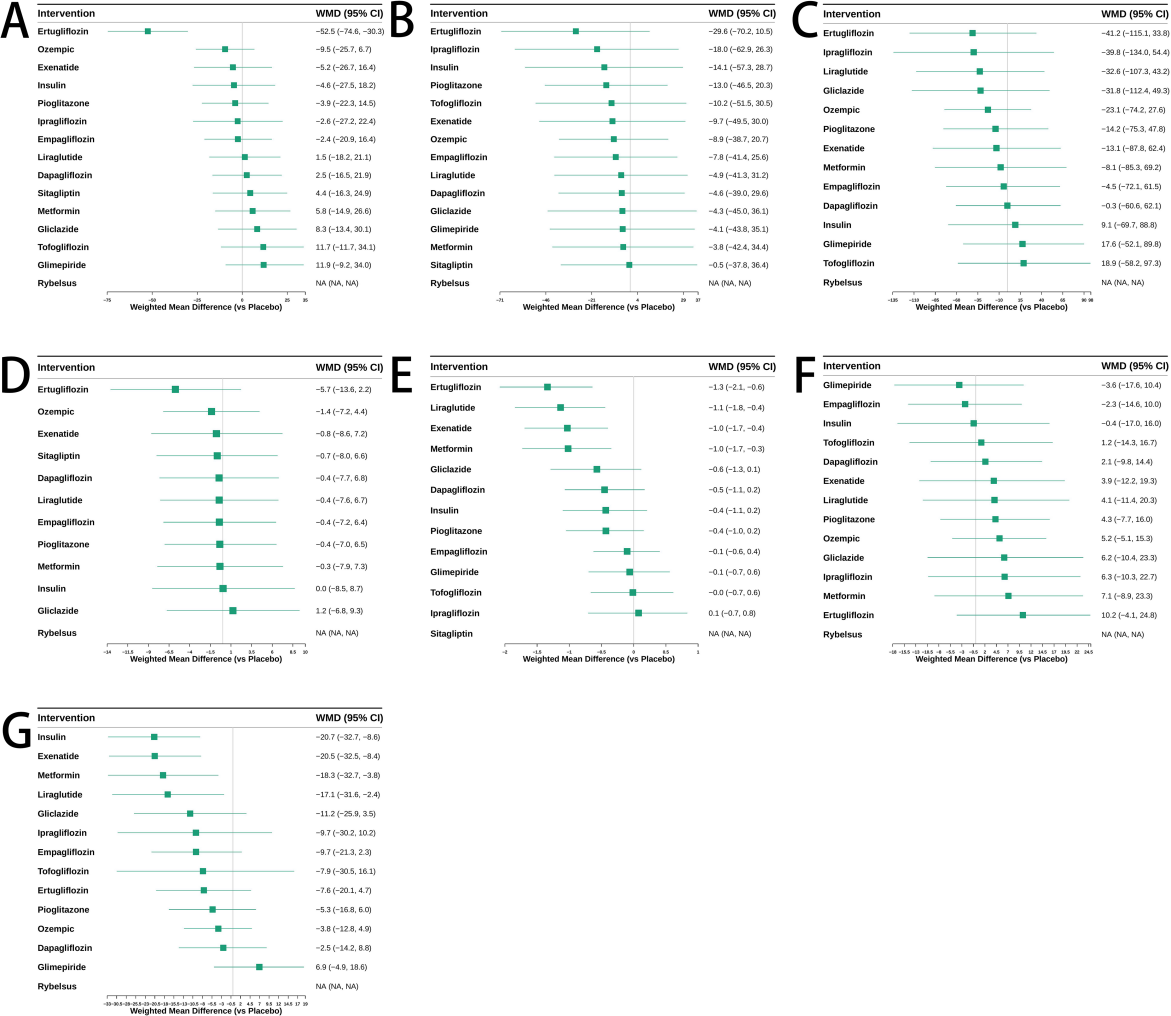
Weighted mean difference (WMD) and 95% confidence intervals for the effects of antidiabetic agents on various outcomes. **(A)** ALT; **(B)** AST; **(C)** Triglycerides; **(D)** BMI; **(E)** HbA1c; **(F)** HDL; **(G)** LDL. Each square represents the WMD for a specific treatment compared to placebo, with horizontal lines indicating the 95% confidence interval. Interventions are ranked in descending order of effect size.

As shown in **Figure 5A**, among the antidiabetic agents evaluated, ertugliflozin appeared to be the most effective in reducing ALT levels in patients with T2DM and MASLD, followed by pioglitazone and metformin. The SUCRA values indicated that these drugs had the highest probabilities of being ranked among the top three treatments. In contrast, glimepiride, tofogliflozin, and gliclazide were among the least effective treatments based on cumulative ranking probabilities. The weighted mean differences (WMDs) presented in **Figure 6A** also supported these findings. Ertugliflozin showed a statistically significant reduction in ALT compared to placebo [WMD = -52.5, 95%CI = (-74.6, -30.3)], while other agents had wide confidence intervals that mostly included zero, indicating non-significant effects. However, these SUCRA rankings should be interpreted cautiously, as several pairwise comparisons were not statistically significant and many credible intervals were wide or overlapped with placebo, reflecting imprecision and sparse data within the network. A detailed comparison of the pairwise effects between treatments on ALT levels is provided in the league table (**Supplementary File Table S3**).

#### AST

As shown in **Figure 5B**, among the antidiabetic agents evaluated, ertugliflozin had the highest probability of being the most effective treatment for reducing AST levels in patients with T2DM and MASLD, followed by pioglitazone and ipragliflozin. In contrast, sitagliptin, rybelsus, and glimepiride ranked among the least effective treatments based on SUCRA values. The WMDs presented in **Figure 6B** were consistent with the SUCRA rankings. Ertugliflozin showed the largest reduction in AST compared to placebo [WMD = -29.6, 95%CI = (-70.2, 10.5)], although the difference was not statistically significant. Other agents, including pioglitazone [WMD = -13.0, 95%CI = (-46.5, 20.3)] and ipragliflozin [WMD = -18.0, 95%CI = (-62.9, 26.3)], showed similar trends but with wide confidence intervals. The detailed pairwise comparisons of the effects of different agents on AST levels are provided in **Supplementary File Table S4**.

#### Triglycerides

Ertugliflozin, liraglutide, and exenatide ranked highest in cumulative probability for reducing triglyceride levels according to the SUCRA plot (**Figure 5C**). Among these, ertugliflozin showed the greatest reduction in triglycerides with a WMD of -41.2 (95% CI: -115.1 to 33.8) compared to placebo, followed by ipragliflozin [-39.8 (-134.0, 54.4)] and liraglutide [-32.6 (-107.3, 43.2)], although none of these differences reached statistical significance (**Figure 6C**). Full pairwise comparisons of treatment effects are available in the **Supplementary File Table S5**.

#### BMI

Among the antidiabetic agents assessed for their effects on body mass index (BMI), ertugliflozin demonstrated the highest probability of being the most effective, as indicated by SUCRA values (**Figure 5D**). This was followed by ozempic and exenatide, while gliclazide, insulin, and metformin ranked among the least effective. According to the WMD results shown in **Figure 6D**, ertugliflozin was associated with the greatest numerical reduction in BMI compared to placebo [WMD = -5.7, 95%CI = (-13.6 to 2.2)], although the difference did not reach statistical significance. All other agents also showed non-significant effects with confidence intervals crossing zero. Full pairwise comparisons of BMI outcomes between treatments are provided in the **Supplementary File Table S6**.

#### HbA1c

Ertugliflozin, liraglutide, and exenatide demonstrated the highest ranking probabilities for HbA1c reduction based on SUCRA values (**Figure 5E**). These agents were followed closely by metformin. In contrast, sitagliptin, tofogliflozin, and ipragliflozin ranked lowest in their ability to reduce HbA1c levels. As shown in the WMD estimates (**Figure 6E**), ertugliflozin [WMD = -1.3, 95%CI = (-2.1, -0.6)], liraglutide [WMD = -1.1, 95%CI = (-1.8, -0.4)], and exenatide [WMD = -1.0, 95%CI = (-1.7, -0.4)] all demonstrated statistically significant reductions in HbA1c compared to placebo. Metformin also showed a significant but slightly smaller effect [WMD = -1.0, 95%CI = (-1.7, -0.3)]. Other interventions produced modest or non-significant changes. Detailed pairwise comparisons of HbA1c outcomes across treatments are available in the **Supplementary File Table S7**.

#### HDL

Ertugliflozin ranked highest in improving HDL levels among all antidiabetic agents based on SUCRA values (**Figure 5F**), followed by metformin and liraglutide. Empagliflozin and glimepiride had the lowest rankings for HDL improvement. The WMD estimates in **Figure 6F** showed that ertugliflozin had the largest numerical increase in HDL compared to placebo [WMD = 10.2, 95%CI = (-4.1, 24.8)], though the difference was not statistically significant. Metformin [WMD = 7.1, 95%CI = (-8.9, 23.3)] and ipragliflozin [WMD = 6.3, 95%CI = (-10.3, 22.7)] also showed positive effects, but none of the treatments achieved significant improvement within the 95% confidence interval. Pairwise comparisons of HDL changes across all included agents are summarized in the **Supplementary File Table S8**.

#### LDL

Liraglutide, exenatide, and metformin ranked as the most effective treatments in reducing LDL cholesterol according to SUCRA estimates (**Figure 5G**). Insulin also showed a high probability of effectiveness, while glimepiride and dapagliflozin were among the least effective. **Figure 6G** shows that insulin [WMD = -20.7; 95% CI (-32.7, -8.6)], exenatide [WMD = -20.5, 95%CI = (-32.5, -8.4)], metformin [WMD = -18.3, 95%CI = (-32.7, -3.8)], and liraglutide [WMD = -17.1, 95%CI = (-31.6, -2.4)] all significantly reduced LDL levels compared to placebo. The effects of other drugs were not statistically significant. Comprehensive comparisons of LDL reduction across all antidiabetic treatments are presented in **Supplementary File Table S9**.

#### LSM

Among the limited data available, empagliflozin showed the highest probability of improving liver stiffness measurement (LSM), followed by pioglitazone. Dapagliflozin ranked last among the evaluated interventions based on SUCRA estimates (**Figure 5H**). Due to the small number of eligible trials reporting LSM outcomes, no WMD estimates could be generated. Full pairwise comparisons are available in the **Supplementary File Table S10**.

### Publication bias

To assess the presence of publication bias, comparison-adjusted funnel plots and Egger’s and Begg’s tests were performed for each outcome. For ALT, both Egger’s (p = 0.0311) and Begg’s (p = 0.0311) tests indicated potential publication bias, as also visually supported by the asymmetry of the funnel plot. In contrast, no significant publication bias was detected for AST (Egger’s p = 0.3506), BMI (p = 0.5021), or LDL, based on both visual inspection and nonsignificant test results. For HbA1c, TG, HDL, and LSM, due to the limited number of studies, statistical tests could not be performed, although funnel plots did not reveal strong asymmetry (**Supplementary File Figure S1**). Therefore, while evidence of potential small-study effects was present in ALT, the remaining outcomes did not demonstrate clear signs of publication bias.

## Discussion

In patients with metabolic dysfunction-associated steatotic liver disease (MASLD) coexisting with type 2 diabetes mellitus (T2DM), the optimal antidiabetic strategy remains an area of active research.

In this network meta-analysis, we compared the efficacy of 15 antidiabetic agents in T2DM patients with MASLD, with particular emphasis on liver function markers, including ALT and AST. Our results demonstrated that ertugliflozin exhibited the most potent effect in reducing ALT levels, followed by pioglitazone and metformin. Similarly, ertugliflozin also ranked highest in improving AST, with pioglitazone and ipragliflozin showing the next best profiles. These differences may be explained by variations in the underlying mechanisms across and within drug classes. For example, although all SGLT2 inhibitors share a common mechanism of promoting urinary glucose excretion, individual agents such as ertugliflozin may produce a greater reduction in intrahepatic triglyceride content due to differences in pharmacokinetic properties, receptor selectivity, or metabolic impact, which could account for its more favorable performance compared with other drugs in the same class.

Among the evaluated agents, ertugliflozin stood out as the most consistently effective across multiple outcomes. In addition to improving ALT and AST levels, it also demonstrated favorable effects in lowering triglyceride levels, reducing body mass index (BMI), increasing HDL-C, decreasing LDL-C, and reducing liver stiffness measurement (LSM). These findings suggest that ertugliflozin may exert hepatoprotective effects through multiple mechanisms. Previous studies have shown that, as a sodium-glucose cotransporter 2 (SGLT2) inhibitor, ertugliflozin can significantly reduce intrahepatic triglyceride (IHTG) content by promoting urinary glucose excretion, thereby alleviating hepatic fat accumulation and improving liver enzyme levels (36). In addition, some studies have reported that ertugliflozin can reduce serum uric acid levels (26), which may be beneficial since elevated uric acid has been implicated in impaired kidney function and may exacerbate liver toxicity in patients with hepatic steatosis (37, 38).

Pioglitazone, a PPAR-γ agonist, also showed beneficial effects, particularly in improving both ALT and AST levels. Previous trials, such as the PIVENS study (39), have highlighted pioglitazone’s histological benefits in MASLD patients, including resolution of steatohepatitis and attenuation of fibrosis. Similarly, a study by Attaran et al. demonstrated that both empagliflozin and pioglitazone significantly improved liver fibrosis (16). The hepatoprotective effects of pioglitazone are likely mediated through increased adiponectin production and enhanced insulin sensitivity in hepatic and adipose tissues (25). Elevated adiponectin levels have been shown to reduce hepatic and systemic insulin resistance and attenuate liver inflammation and fibrosis, thereby contributing to the overall improvement of MASLD (40).

While agents such as glimepiride, sitagliptin, and tofogliflozin ranked lower in their efficacy for improving liver-related outcomes in this analysis, these medications may still play a role in specific clinical contexts. For example, sitagliptin, a DPP-4 inhibitor, is generally well tolerated and has a low risk of hypoglycemia, making it a suitable option for elderly patients or those with renal impairment (41, 42). Similarly, glimepiride, though less effective in reducing hepatic biomarkers, remains a cost-effective and widely accessible sulfonylurea that may be considered in resource-limited settings. Therefore, when developing treatment strategies, it is important to consider not only drug efficacy but also individual patient factors such as comorbidities, tolerability, and economic burden.

To our knowledge, this is the most comprehensive and up-to-date network meta-analysis evaluating the relative efficacy of antidiabetic agents in patients with T2DM and MASLD. However, several limitations must be acknowledged. First, heterogeneity among the included studies—including variations in drug dosages, treatment durations, study populations, and follow-up periods—may have influenced the reliability of the pooled estimates. Second, the geographic and ethnic representation of the included studies was limited, as the majority of trials were conducted in Asian countries, with only a few originating from non-Asian regions. This lack of diversity may restrict the generalizability of our findings to broader populations, particularly Western or multiethnic cohorts. Third, signs of potential publication bias were detected, particularly in the analysis of ALT, where both Egger’s and Begg’s tests suggested asymmetry, possibly reflecting small-study effects or selective reporting. Given that ALT was a primary outcome, this potential publication bias may have influenced the pooled effect estimates and should be considered when interpreting the robustness of the results. Fourth, the mechanisms of action of some antidiabetic agents on liver-related outcomes remain incompletely understood, and additional mechanistic studies are needed to clarify these effects. Fifth, the SUCRA-based rankings should be interpreted cautiously, as several estimates were imprecise and may be influenced by sparse data, which could introduce uncertainty or bias into the probabilistic ranking results. Finally, our analysis was limited to the efficacy of individual monotherapies; however, in clinical settings, combination therapies are frequently used and may provide additive or synergistic benefits. Future trials should evaluate the comparative effectiveness of combination regimens and support the development of individualized treatment strategies for patients with T2DM and MASLD.

## Conclusion

In conclusion, our findings suggest that ertugliflozin appears to be among the most effective treatments for improving liver function in patients with metabolic dysfunction-associated steatotic liver disease (MASLD) and type 2 diabetes mellitus (T2DM). Given its favorable impact on ALT, AST, and other metabolic parameters, ertugliflozin shows strong potential as a preferred treatment option in this population. However, further high-quality randomized controlled trials are warranted to validate these findings and to explore the long-term hepatic benefits of SGLT-2 inhibitors.
