# Supplementary material for: Comparison of the efficacy of antidiabetic agents in type 2 diabetes with MASLD: a network meta-analysis
Source: Front Endocrinol (Lausanne). 2026 Jan 7;16:1659740. doi: 10.3389/fendo.2025.1659740 (PMC12819300; doi:10.3389/fendo.2025.1659740)

| **Table S1.** **details of search strategy (from database inception to April 10, 2025)** | | |
| --- | --- | --- |
| **No.** | **Search Strategies** | **Results** |
| **PubMed** | | |
| #1 | "Non-alcoholic Fatty Liver Disease"[Mesh] | 28,838 |
| #2 | (((((("Non-alcoholic Fatty Liver Disease"[Mesh]) OR (fatty liver[Title/Abstract])) OR (NAFLD[Title/Abstract])) OR (MAFLD[Title/Abstract])) OR (Steatohepatitis[Title/Abstract])) OR (steatotic liver[Title/Abstract])) OR (steatotic liver disease[Title/Abstract]) OR (MASLD[Title/Abstract]) OR (MASH[Title/Abstract]) OR (Steatosis of Liver[Title/Abstract]) OR (MASL[Title/Abstract]) | 69,964 |
| #3 | "Diabetes Mellitus, Type 2"[Mesh] | 189,424 |
| #4 | ("Diabetes Mellitus, Type 2"[Mesh] OR "Diabetes"[Title/Abstract] OR "Type 2 Diabetes Mellitus"[Title/Abstract] OR T2DM[Title/Abstract]) | 763,275 |
| #5 | "Hypoglycemic Agents"[Mesh] | 95,666 |
| #6 | ("Hypoglycemic Agents"[Mesh] OR Dapagliflozin[Title/Abstract] OR Empagliflozin[Title/Abstract] OR Ertugliflozin[Title/Abstract] OR Tofogliflozin[Title/Abstract] OR Ipragliflozin[Title/Abstract] OR Liraglutide[Title/Abstract] OR Exenatide[Title/Abstract] OR Beinaglutide[Title/Abstract] OR Rybelsus[Title/Abstract] OR Ozempic[Title/Abstract] OR Sitagliptin[Title/Abstract] OR Insulin glargine[Title/Abstract] OR Glimepiride[Title/Abstract] OR Gliclazide[Title/Abstract] OR Pioglitazone[Title/Abstract] OR Metformin[Title/Abstract]) | 124,463 |
| #7 | #2 AND #4 AND #6 | 1,428 |
| **Embase** | | |
| #1 | ‘nonalcoholic fatty liver’/exp | 84,614 |
| #2 | 'Non-alcoholic Fatty Liver Disease':ab,ti OR 'fatty liver':ab,ti OR 'NAFLD':ab,ti OR 'MAFLD':ab,ti OR 'Steatohepatitis':ab,ti OR 'steatotic liver':ab,ti OR 'steatotic liver disease':ab,ti OR 'MASLD':ab,ti OR 'MASH':ab,ti OR 'Steatosis of Liver':ab,ti OR 'MASL':ab,ti | 104,673 |
| #3 | ‘Diabetes Mellitus, Type 2’/exp | 404,791 |
| #4 | 'diabetes':ab,ti OR 'type 2 diabetes mellitus':ab,ti OR t2dm:ab,ti | 1,128,493 |
| #5 | ‘Hypoglycemic Agents’/exp | 694,319 |
| #6 | 'dapagliflozin':ab,ti OR 'empagliflozin':ab,ti OR 'ertugliflozin':ab,ti OR 'tofogliflozin':ab,ti OR 'ipragliflozin':ab,ti OR 'liraglutide':ab,ti OR 'exenatide':ab,ti OR 'beinaglutide':ab,ti OR 'rybelsus':ab,ti OR 'ozempic':ab,ti OR 'sitagliptin':ab,ti OR 'insulin glargine':ab,ti OR 'glimepiride':ab,ti OR 'gliclazide':ab,ti OR 'pioglitazone':ab,ti OR 'metformin':ab,ti | 90,848 |
| #7 | 'randomized controlled trial':ab,ti OR randomized:ab,ti OR placebo:ab,ti OR rct:ab,ti | 1,305,551 |
| #8 | (#1 OR #2) AND (#3 OR #4) AND (#5 OR #6) AND #7 | 838 |
| **C****ochrane Library** | | |
| #1 | MeSH descriptor: [Non-alcoholic Fatty Liver Disease] explode all trees | 3,673 |
| #2 | (fatty liver):ab,ti,kw OR (NAFLD):ab,ti,kw OR (MAFLD):ab,ti,kw OR (Steatohepatitis):ab,ti,kw OR (steatotic liver):ab,ti,kw OR (steatotic liver disease):ab,ti,kw OR (MASLD):ab,ti,kw OR (MASH):ab,ti,kw OR (Steatosis of Liver):ab,ti,kw OR (MASL):ab,ti,kw | 8,634 |
| #3 | MeSH descriptor: [Diabetes Mellitus, Type 2] explode all trees | 75,280 |
| #4 | (diabetes):ab,ti,kw OR (type 2 diabetes mellitus):ab,ti,kw OR (T2DM):ab,ti,kw | 112,273 |
| #5 | MeSH descriptor: [Hypoglycemic Agents] explode all trees | 11,955 |
| #6 | (dapagliflozin):ab,ti,kw OR (empagliflozin):ab,ti,kw OR (ertugliflozin):ab,ti,kw OR (tofogliflozin):ab,ti,kw OR (ipragliflozin):ab,ti,kw OR (liraglutide):ab,ti,kw OR (exenatide):ab,ti,kw OR (beinaglutide):ab,ti,kw OR (rybelsus):ab,ti,kw OR (ozempic):ab,ti,kw OR (sitagliptin):ab,ti,kw OR (insulin glargine):ab,ti,kw OR (glimepiride):ab,ti,kw OR (gliclazide):ab,ti,kw OR (pioglitazone):ab,ti,kw OR (metformin):ab,ti,kw | 25,717 |
| #7 | (#1 OR #2) AND (#3 OR #4) AND (#5 OR #6) | 713 |
| **Web of science** | | |
| #1 | Non-alcoholic Fatty Liver Disease OR fatty liver OR NAFLD OR MAFLD OR Steatohepatitis OR steatotic liver OR steatotic liver disease OR MASLD OR MASH OR Steatosis of Liver OR MASL | 265,424 |
| #2 | "Diabetes Mellitus, Type 2" OR "Type 2 Diabetes Mellitus" OR Diabetes OR T2DM | 1,478,538 |
| #3 | "Hypoglycemic Agents" OR Dapagliflozin OR Empagliflozin OR Ertugliflozin OR Tofogliflozin OR Ipragliflozin OR Liraglutide OR Exenatide OR Beinaglutide OR Rybelsus OR Ozempic OR Sitagliptin OR "Insulin glargine" OR Glimepiride OR Gliclazide OR Pioglitazone OR Metformin | 172,000 |
| #4 | "randomized controlled trial" OR randomized OR placebo OR RCT | 1,739,466 |
| #5 | #1 AND #2 AND #3 AND #4 | 889 |

| **Table S2. Reason description for exclusion of 27 studies** | | | |
| --- | --- | --- | --- |
| **No.** | **First author** | **Publication year** | **Reason for exclusion** |
| 1 | A Nar | 2009 | Combination therapy; single-drug effect not isolatable |
| 2 | Mohammad S Kuchay | 2020 | Combination therapy; single-drug effect not isolatable |
| 3 | Abbas Ali Sangoun | 2021 | Combination therapy; single-drug effect not isolatable |
| 4 | Srinivasan Dasarathy | 2015 | Lack of relevant data |
| 5 | Yukihiro Bando | 2017 | Lack of relevant data |
| 6 | Niina Matikainen | 2018 | Lack of relevant data |
| 7 | Maurice B Bizino | 2020 | Lack of relevant data |
| 8 | Susrichit Phrueksotsai | 2021 | Lack of relevant data |
| 9 | Hirokazu Takahashi | 2021 | Lack of relevant data |
| 10 | Mengran Shi | 2023 | Lack of relevant data |
| 11 | Yujuan Fan | 2024 | Lack of relevant data |
| 12 | An Tang | 2015 | No outcome of interest |
| 13 | Anna Vanderheiden | 2016 | No outcome of interest |
| 14 | Ryotaro Bouchi | 2017 | No outcome of interest |
| 15 | Aditi R Saxena | 2023 | No outcome of interest |
| 16 | Mona S Abdel Monem | 2025 | No outcome of interest |
| 17 | Kiichi Hirayama | 2025 | No outcome of interest |
| 18 | Meng-Tzu Weng | 2025 | No outcome of interest |
| 19 | Jingxuan Lian | 2021 | Reviews |
| 20 | Yuan Zhu | 2021 | Reviews |
| 21 | Manjun Deng | 2023 | Reviews |
| 22 | Yuan-Yuan Xu | 2024 | Reviews |
| 23 | Laya Hooshmand Gharabagh | 2024 | Reviews |
| 24 | Hua Duan | 2025 | Reviews |
| 25 | Lingyan Liu | 2025 | Reviews |
| 26 | Xiao-Long Deng | 2017 | Unable to get full-text |
| 27 | Eugene Han | 2021 | Unable to get full-text |
| **Excluded studies for the table[1-27]**  1. Nar, A. and O. Gedik, *The effect of metformin on leptin in obese patients with type 2 diabetes mellitus and nonalcoholic fatty liver disease.* Acta Diabetol, 2009. **46**(2): p. 113-8.  2. Kuchay, M.S., et al., *Effect of dulaglutide on liver fat in patients with type 2 diabetes and NAFLD: randomised controlled trial (D-LIFT trial).* Diabetologia, 2020. **63**(11): p. 2434-2445.  3. Sangouni, A.A., Z. Orang, and H. Mozaffari-Khosravi, *Effect of omega-3 supplementation on fatty liver and visceral adiposity indices in diabetic patients with non-alcoholic fatty liver disease: A randomized controlled trial.* Clin Nutr ESPEN, 2021. **44**: p. 130-135.  4. Dasarathy, S., et al., *Double-blind randomized placebo-controlled clinical trial of omega 3 fatty acids for the treatment of diabetic patients with nonalcoholic steatohepatitis.* J Clin Gastroenterol, 2015. **49**(2): p. 137-44.  5. Bando, Y., et al., *The effects of ipragliflozin on the liver-to-spleen attenuation ratio as assessed by computed tomography and on alanine transaminase levels in Japanese patients with type 2 diabetes mellitus.* Diabetol Int, 2017. **8**(2): p. 218-227.  6. Matikainen, N., et al., *Liraglutide treatment improves postprandial lipid metabolism and cardiometabolic risk factors in humans with adequately controlled type 2 diabetes: A single-centre randomized controlled study.* Diabetes Obes Metab, 2019. **21**(1): p. 84-94.  7. Bizino, M.B., et al., *Placebo-controlled randomised trial with liraglutide on magnetic resonance endpoints in individuals with type 2 diabetes: a pre-specified secondary study on ectopic fat accumulation.* Diabetologia, 2020. **63**(1): p. 65-74.  8. Phrueksotsai, S., et al., *The effects of dapagliflozin on hepatic and visceral fat in type 2 diabetes patients with non-alcoholic fatty liver disease.* J Gastroenterol Hepatol, 2021. **36**(10): p. 2952-2959.  9. Takahashi, H., et al., *Ipragliflozin Improves the Hepatic Outcomes of Patients With Diabetes with NAFLD.* Hepatol Commun, 2022. **6**(1): p. 120-132.  10. Shi, M., et al., *Effect of dapagliflozin on liver and pancreatic fat in patients with type 2 diabetes and non-alcoholic fatty liver disease.* J Diabetes Complications, 2023. **37**(10): p. 108610.  11. Fan, Y., et al., *Efficacy of beinaglutide in the treatment of hepatic steatosis in type 2 diabetes patients with nonalcoholic fatty liver disease: A randomized, open-label, controlled trial.* Diabetes Obes Metab, 2024. **26**(2): p. 772-776.  12. Tang, A., et al., *Effects of Insulin Glargine and Liraglutide Therapy on Liver Fat as Measured by Magnetic Resonance in Patients With Type 2 Diabetes: A Randomized Trial.* Diabetes Care, 2015. **38**(7): p. 1339-46.  13. Vanderheiden, A., et al., *Mechanisms of Action of Liraglutide in Patients With Type 2 Diabetes Treated With High-Dose Insulin.* J Clin Endocrinol Metab, 2016. **101**(4): p. 1798-806.  14. Bouchi, R., et al., *Reduction of visceral fat by liraglutide is associated with ameliorations of hepatic steatosis, albuminuria, and micro-inflammation in type 2 diabetic patients with insulin treatment: a randomized control trial.* Endocr J, 2017. **64**(3): p. 269-281.  15. Saxena, A.R., et al., *A phase 2a, randomized, double-blind, placebo-controlled, three-arm, parallel-group study to assess the efficacy, safety, tolerability and pharmacodynamics of PF-06835919 in patients with non-alcoholic fatty liver disease and type 2 diabetes.* Diabetes Obes Metab, 2023. **25**(4): p. 992-1001.  16. Abdel Monem, M.S., et al., *Efficacy and safety of dapagliflozin compared to pioglitazone in diabetic and non-diabetic patients with non-alcoholic steatohepatitis: A randomized clinical trial.* Clin Res Hepatol Gastroenterol, 2025. **49**(3): p. 102543.  17. Hirayama, K., et al., *Effects of the SGLT2 inhibitor ipragliflozin and metformin on hepatic steatosis and liver fibrosis: Sub-analysis of a randomized controlled study.* Diabetes Obes Metab, 2025. **27**(4): p. 2059-2069.  18. Weng, M.T., et al., *Effects of dapagliflozin on liver steatosis in patients with nonalcoholic fatty liver disease: a randomized controlled trial.* Hepatol Int, 2025. **19**(2): p. 405-414.  19. Lian, J. and J. Fu, *Pioglitazone for NAFLD Patients With Prediabetes or Type 2 Diabetes Mellitus: A Meta-Analysis.* Front Endocrinol (Lausanne), 2021. **12**: p. 615409.  20. Zhu, Y., et al., *Efficacy and Safety of GLP-1 Receptor Agonists in Patients With Type 2 Diabetes Mellitus and Non-Alcoholic Fatty Liver Disease: A Systematic Review and Meta-Analysis.* Front Endocrinol (Lausanne), 2021. **12**: p. 769069.  21. Deng, M., et al., *Comparative effectiveness of multiple different treatment regimens for nonalcoholic fatty liver disease with type 2 diabetes mellitus: a systematic review and Bayesian network meta-analysis of randomised controlled trials.* BMC Med, 2023. **21**(1): p. 447.  22. Xu, Y.Y., et al., *Meta-analysis of the clinical efficacy of liraglutide in treating type 2 diabetes mellitus complicated with non-alcoholic fatty liver disease.* Endocr J, 2024. **71**(9): p. 881-894.  23. Hooshmand Gharabagh, L., et al., *Comparison between the effect of Empagliflozin and Pioglitazone added to metformin in patients with type 2 diabetes and nonalcoholic fatty liver disease.* Clin Res Hepatol Gastroenterol, 2024. **48**(3): p. 102279.  24. Duan, H. and F. Chen, *Efficacy of dapagliflozin to treat nonalcoholic fatty liver disease in patients with type 2 diabetes: A meta-analysis.* Medicine (Baltimore), 2025. **104**(1): p. e40836.  25. Liu, L., et al., *Comparison of efficacy and safety of pioglitazone and SGLT2 inhibitors in treating Asian patients in MASLD associated with type 2 diabetes: A meta-analysis.* J Diabetes Complications, 2025. **39**(4): p. 108998.  26. Deng, X.L., et al., *Short article: A randomized-controlled study of sitagliptin for treating diabetes mellitus complicated by nonalcoholic fatty liver disease.* Eur J Gastroenterol Hepatol, 2017. **29**(3): p. 297-301.  27. Han, E., et al., *Efficacy and safety of evogliptin in patients with type 2 diabetes and non-alcoholic fatty liver disease: A multicentre, double-blind, randomized, comparative trial.* Diabetes Obes Metab, 2022. **24**(4): p. 752-756. | | | |

**Table S3** Network analysis results of comparison between different drugs in ALT

**Table S4** Network analysis results of comparison between different drugs in AST

**Table S5** Network analysis results of comparison between different drugs in Triglycerides

**Table S6** Network analysis results of comparison between different drugs in BMI

**Table S7** Network analysis results of comparison between different drugs in HbA1c

**Table S8** Network analysis results of comparison between different drugs in HDL

**Table S9** Network analysis results of comparison between different drugs in LDL

**Table S10** Network analysis results of comparison between different drugs in LSM

**Fig.S1** Funnel plot on publication bias


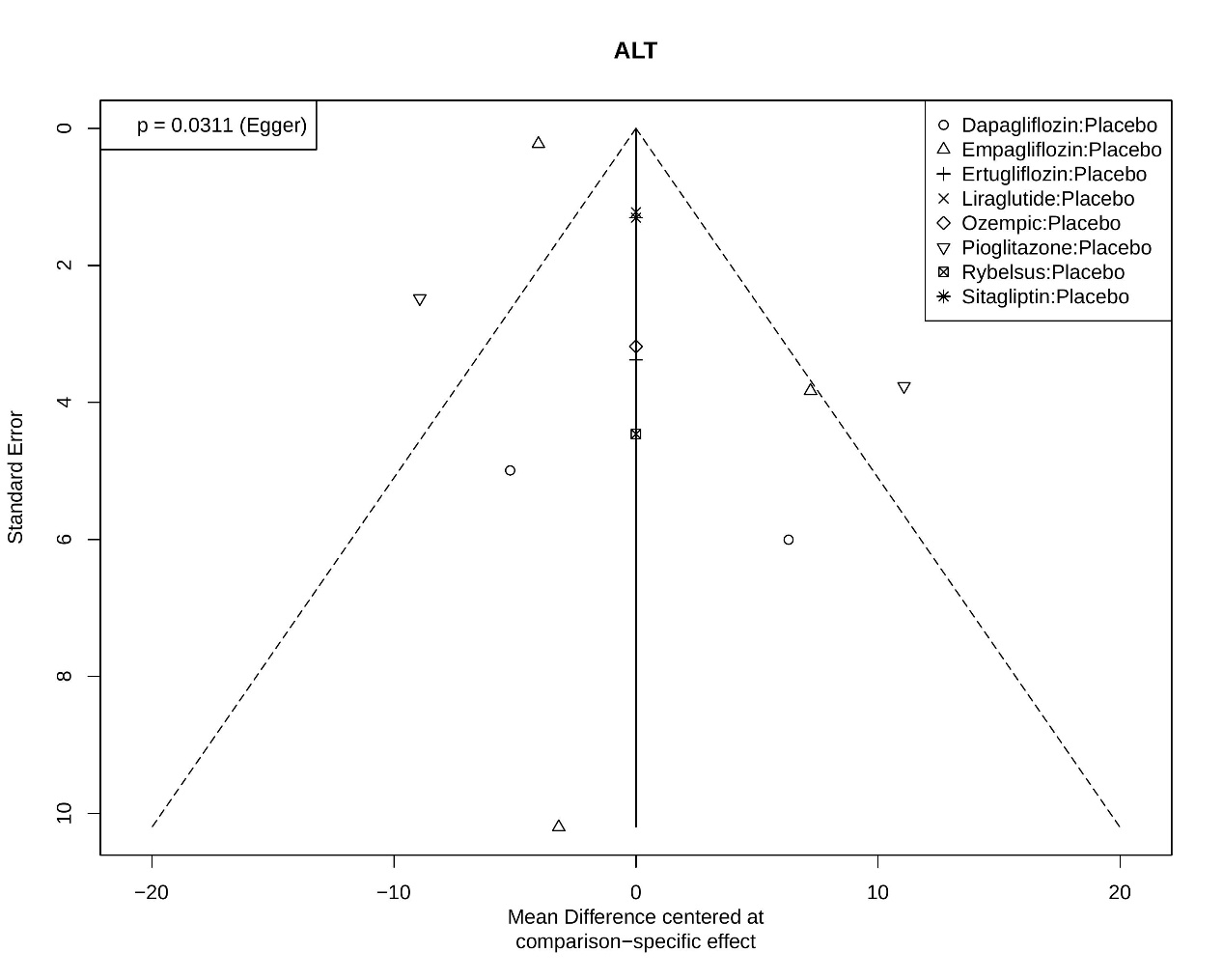


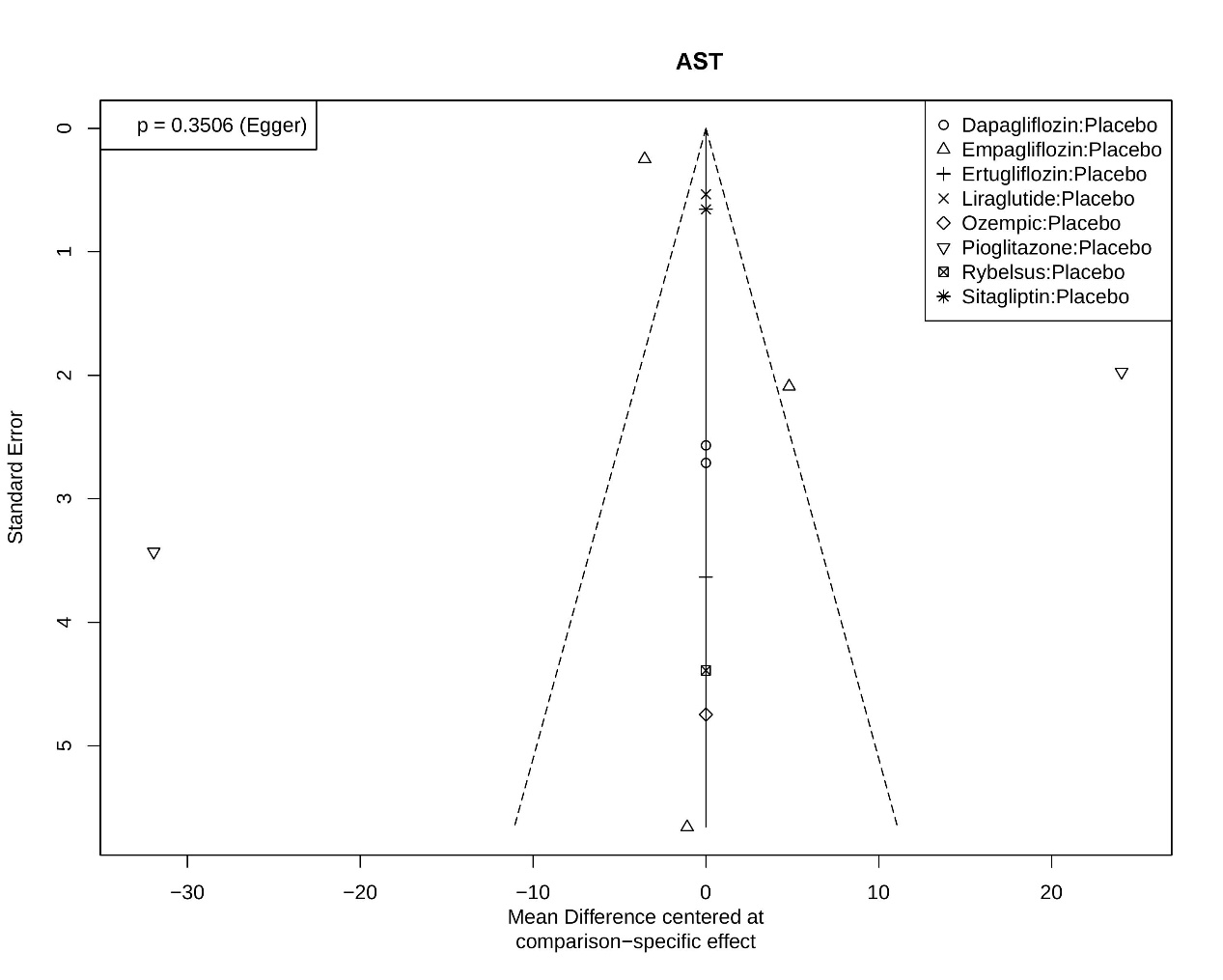


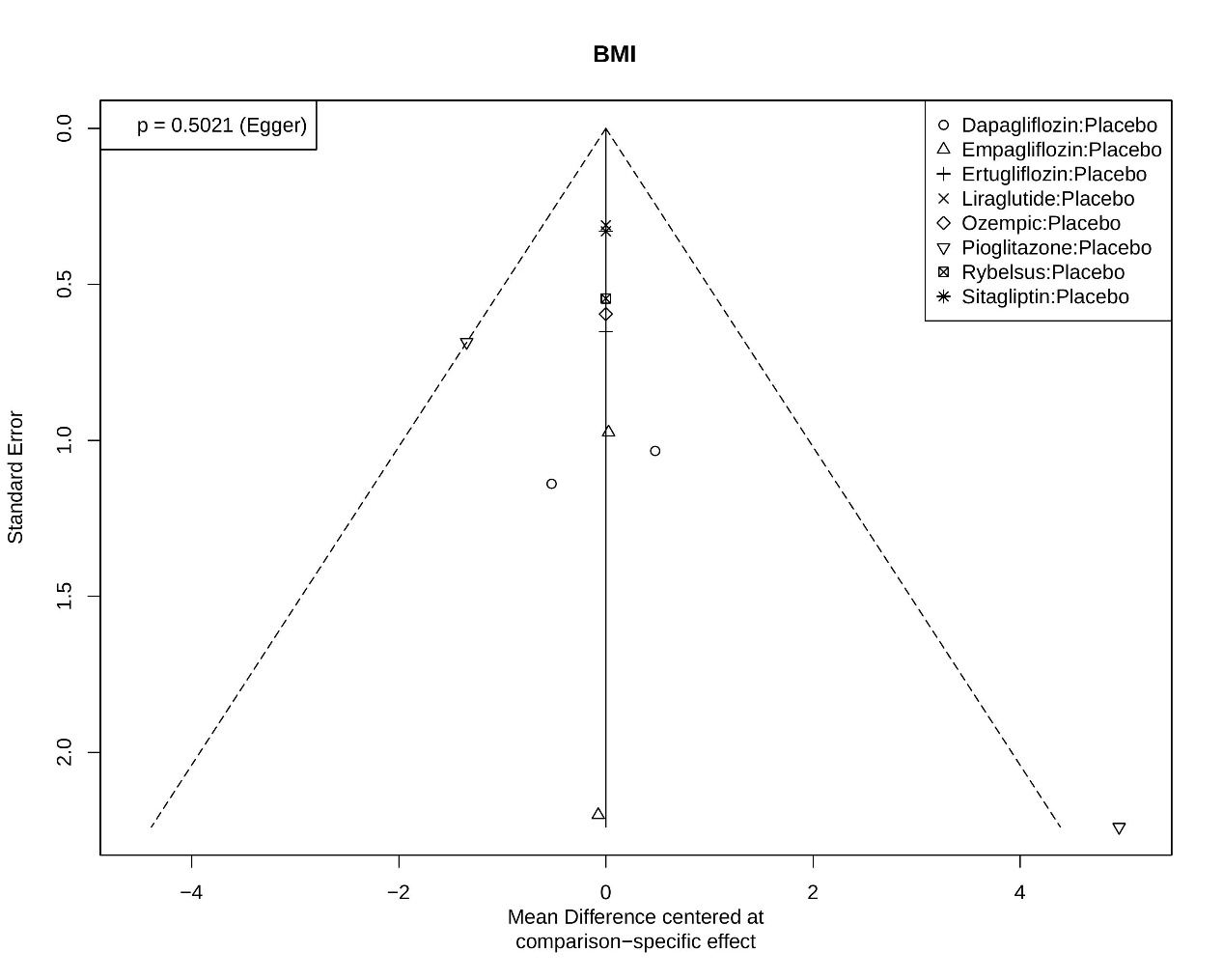


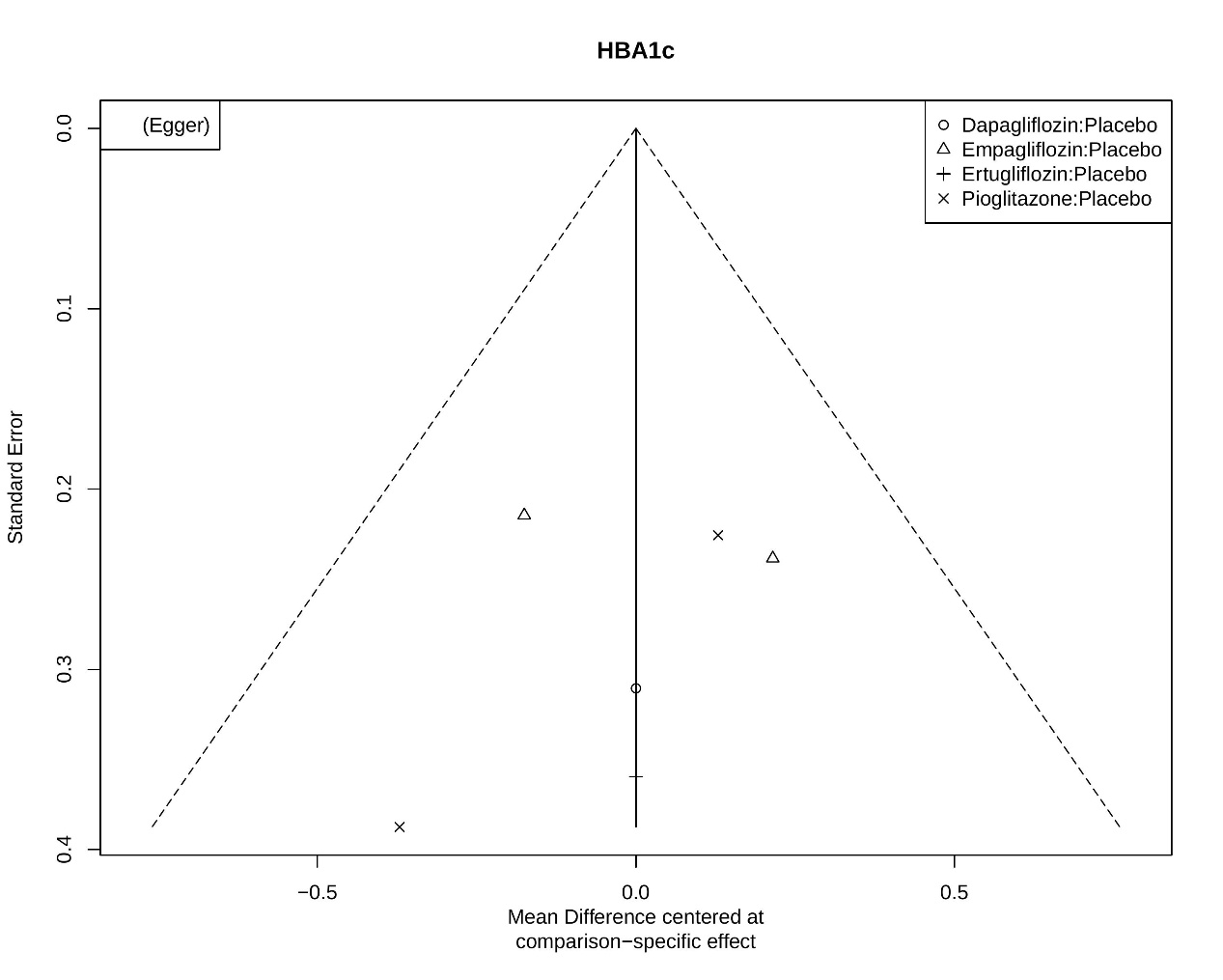


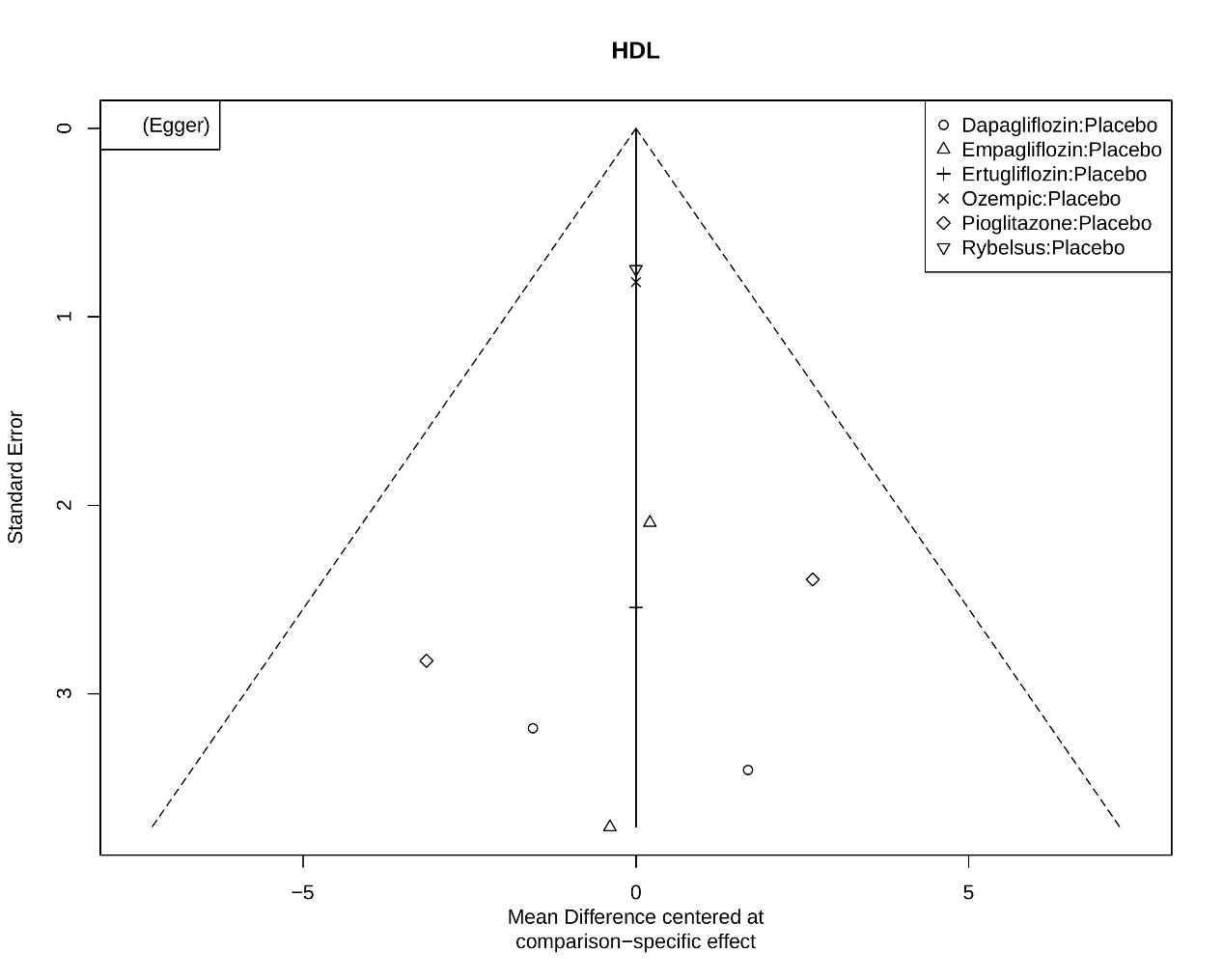


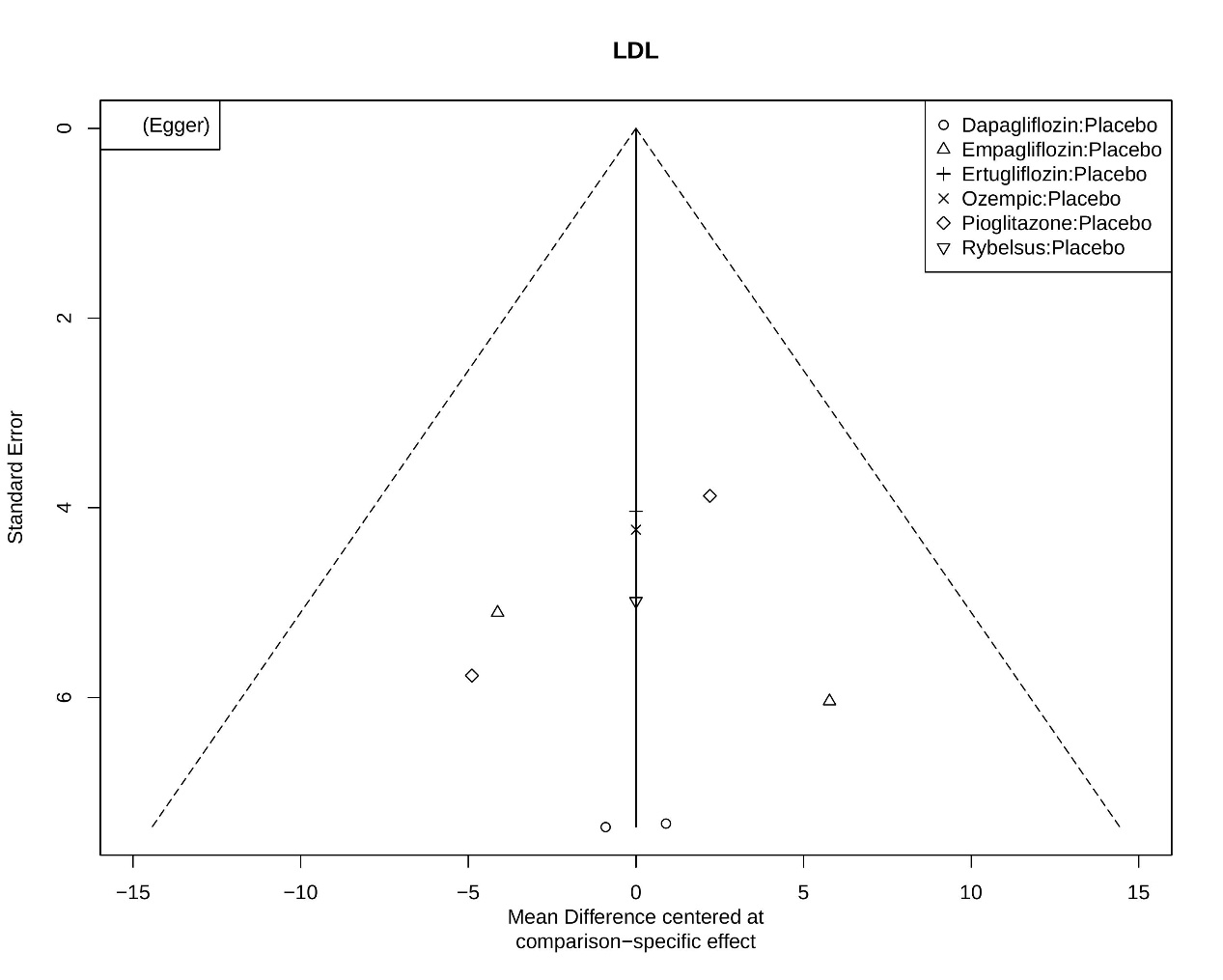


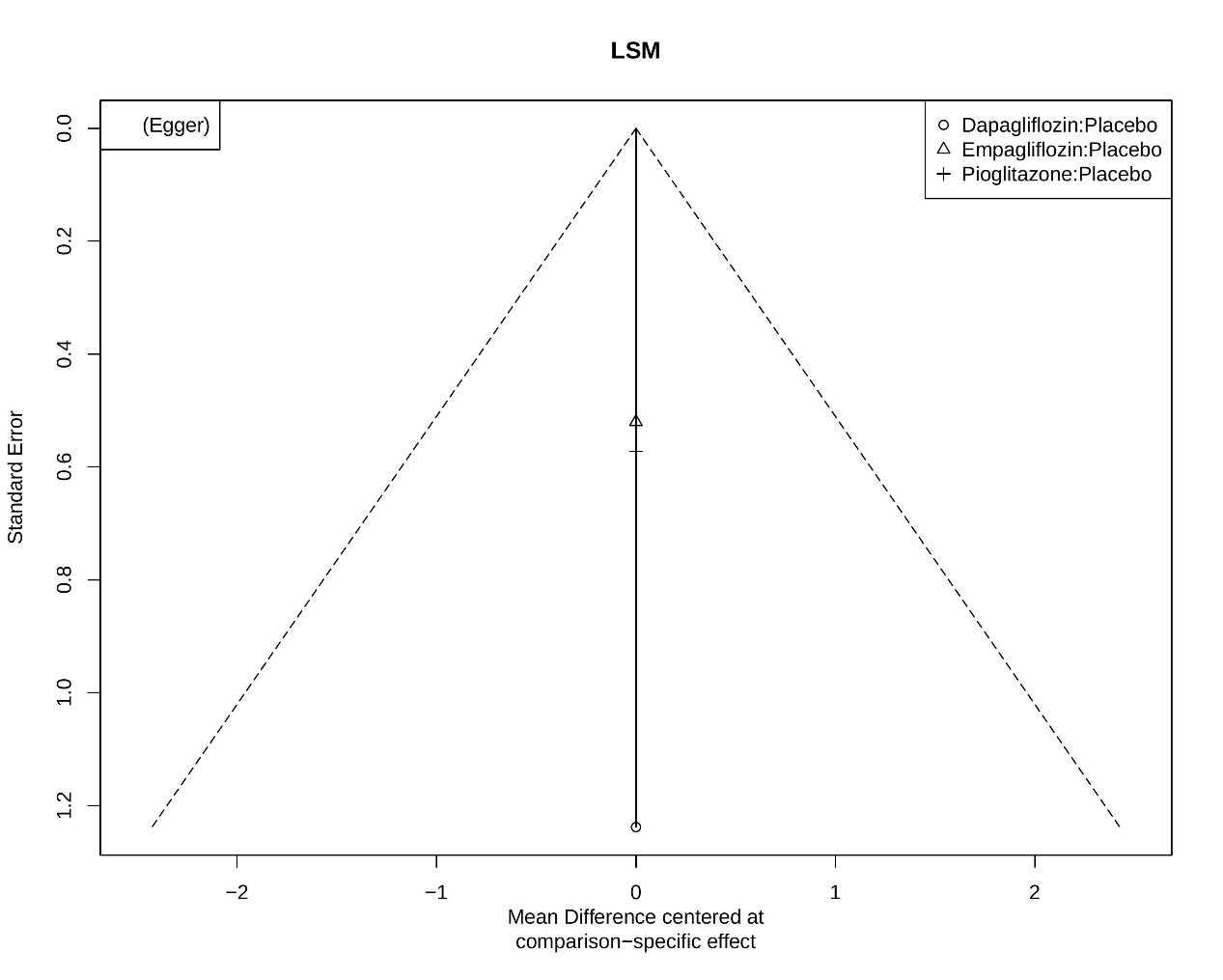


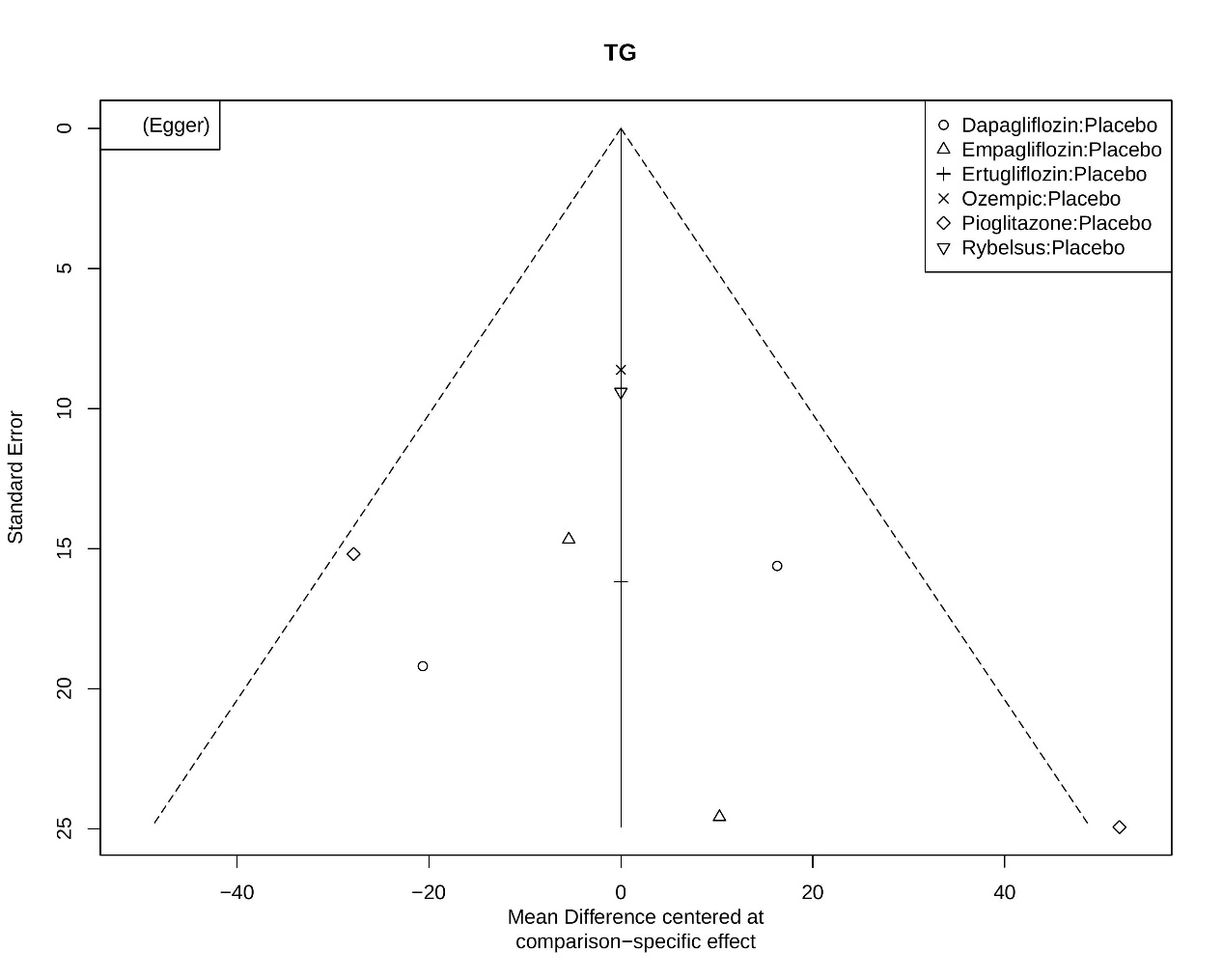

Supplement: Supplementary file 3 [file Table3.docx]
